# Animal models to study the pathogenesis and novel therapeutics of oral lichen planus

**DOI:** 10.3389/froh.2024.1405245

**Published:** 2024-05-09

**Authors:** Youngnim Choi

**Affiliations:** Department of Immunology and Molecular Microbiology in Dental Science, Seoul National University School of Dentistry and Dental Research Institute, Seoul, Republic of Korea

**Keywords:** oral lichen planus, animal model, validity, pathogenesis, therapeutics

## Abstract

Oral lichen planus (OLP) is a prevalent oral mucosal disease characterized by an unknown etiology and a complex pathogenesis. Patients with OLP endure a chronic course marked by alternating non-erosive and erosive lesions, with no definitive cure currently available. Particularly challenging is the treatment of recalcitrant erosive OLP, highlighting an urgent need for therapies targeting specific pathogenic pathways. In diseases like OLP, where the etiopathogenesis is intricate and elusive, animal models are indispensable for hypothesis testing and elucidating disease mechanisms. To date, only three animal models for oral lichenoid lesions have been reported in the literature. This Perspective paper evaluates these existing models, along with a novel OLP mouse model introduced at the 3rd International Conference on Oral Mucosal Immunity and Microbiome. The validity of these models is critically assessed, and their potential future applications in advancing our understanding of OLP are discussed.

## 1 Introduction

Oral lichen planus (OLP) is a common oral mucosal disease of unknown etiology, affecting approximately 0.5%–1.7% of the general population (1). Clinically, OLP is characterized by bilateral white reticular or plaque-like lesions on the buccal mucosae, tongue, or gingivae, often accompanied by atrophic (erythematous), erosive (ulcerative), bullous, or papular lesions. OLP lesions can be broadly categorized into erosive (atrophic/erosive/bullous) and non-erosive (reticular/plaque/papular) types. Non-erosive lesions are relatively asymptomatic, while erosive lesions cause discomfort, a burning sensation, or pain (1).

The American Academy of Oral and Maxillofacial Pathology, in its 2016 position paper, defined the histopathologic criteria for OLP diagnosis as (i) a band-like or patchy predominantly lymphocytic infiltrate in the subepithelial lamina propria, (ii) hydropic degeneration of the basal cell layer, (iii) lymphocytic exocytosis (migration of lymphocytes into the epithelium), (iv) absence of epithelial dysplasia, and (v) absence of verrucous epithelial architectural changes (2). Hydropic degeneration of the basal cells often co-occurs with colloid (also called as cytoid, or Civatte) bodies and the dissolution of the basement membrane (3). Additional histologic features may include hyperparakeratosis, acanthosis, saw-tooth rete pegs, and fibrinous eosinophilic deposits at the epithelial-lamina propria interface (2, 3).

Several oral lichenoid lesions (OLLs) clinically and/or histologically mimic OLP but have known etiologies, except for chronic ulcerative stomatitis. OLLs with known etiologies include mucous membrane pemphigoid caused by autoantibodies against the basement membrane, oral lichenoid drug reaction caused by systemic medications, amalgam-associated oral lichenoid contact reaction (OLCR) from direct contact with dental amalgam, cinnamon-associated OLCR from direct contact with cinnamon, and chronic graft-vs.-host disease (GvHD) following allogeneic hematopoietic stem cell transplantation (2, 3).

Despite extensive research, the etiology of OLP remains unknown. Proposed triggers include stress, hypothyroidism, systemic medications, trauma, trace element deficiency, food allergy, and microbial infections (2–5). The disease is primarily mediated by T lymphocytes, including both CD4^+^ and CD8^+^ cells, with a significant role for CD8^+^ cytotoxic T cells in basal cell hydropic degeneration (5). Additionally, the involvement of diverse Th cell subsets, including Th1, Th2, Th17, Th9, and Treg, in OLP pathology, has been proposed (5). However, the specific roles of these subsets in different clinical types and during disease quiescence vs. flare-up phases, as well as the precise nature of antigens activating T cells, remain inconclusive (5).

Currently, there is no cure for OLP, and patients experience a chronic, relapsing-remitting course alternating between non-erosive and erosive lesions (6). The World Health Organization designated OLP as an oral potentially malignant disorder. A recent meta-analysis reported OLP's malignant transformation rate as 1.14%, with atrophic-erosive lesions posing a fourfold higher risk than reticular lesions (7). Management primarily focuses on alleviating discomfort from erosive lesions and monitoring for malignant transformation. First-line therapy includes corticosteroids (topical, intralesional, systemic), with additional treatment options, such as hydroxychloroquine, mycophenolate mofetil, methotrexate, retinoids, azathioprine, calcineurin inhibitors, JAK/STAT inhibitors, and biologics targeting TNF*α*, CD2, IL17, IL12/23, or IL23, for recalcitrant erosive OLP (6, 8). However, the effectiveness of these therapies has not been proven through double-blind randomized clinical trials, underscoring the need for the development of treatments targeting specific pathogenic pathways in OLP.

## 2 The role of animal models in medical research

Animal models are indispensable in biomedical research, significantly contributing to our understanding of disease mechanisms and the development of novel treatments. They are essential for studying complex biological processes within a living organism, often impossible with *in vitro* systems alone. While human studies provide more biologically relevant results, conflicting outcomes are common due to the inability to control all variables; most human studies are cross-sectional, limiting causal inferences. Animal models offer critical insights into human disease pathophysiology, elucidating how diseases develop and progress. The greatest advantage of animal model studies is the ability to address mechanistic questions and test cause-and-effect relationships within a controlled environment (9). For diseases like OLP, with complex and elusive etiopathogenesis, animal models are vital for testing hypotheses on disease mechanisms. Furthermore, animal models are crucial in assessing new therapeutic agents’ efficacy and safety before human clinical trials.

## 3 Criteria for the validation of OLP animal models

The validation of animal models relies on the principles of predictive, face, and construct validity (Table 1). These criteria, originally developed for validating depression animal models (10), are now universally applied in medical research involving animal models.

• Predictive Validity: This refers to how well an animal model’s response to treatments predicts therapeutic response in humans. For OLP, animal models should exhibit a positive response to topical corticosteroids, mirroring the primary therapy used in human patients.
• Face Validity: This pertains to phenotypic similarity, assessing how accurately the animal model replicates the symptoms and pathology of human disease. An ideal OLP model should exhibit key features of human OLP, including multiple symmetric distribution of reticular/erosive lesions on the oral mucosa, band-like or patch subepithelial lymphocytic infiltrate, intra-epithelial T cells, and hydropic degeneration of the basal cell layer with colloid bodies.
• Construct Validity: This relates to mechanistic similarity and how well the method used to induce the disease in animals reflects human disease etiopathogenesis. For OLP, construct validity could relate to etiological factors or immunological mechanisms. While definitive theories on OLP etiopathogenesis are limited, the method used in animal models should induce at least one of the face validities for OLP. Additionally, various hypotheses regarding disease mechanisms can be tested.

**Table 1:** OLP animal models and their validity.

		Shiohara et al. (11)	Thomas et al. (12)	Dunsche et al. (13)	Phuc et al. (14)
Induction method		s.c. injection of Ia-reactive CD4^+^ T-cell clone	i.v. injection of spleen cells: Lew (RT1^l^) → Lew/Da (RTl^av1^) F^1^	Contact of buccal mucosa with amalgam block	Oral mucosal infection with *E. coli* infection under zinc deficiency
Predictive Validity	Response to corticosteroids	Not tested	Not tested	Not tested	Not tested
Face Validity	White reticular/red erosive lesions	Irrelevant	No	White plaque-like lesion at amalgam-exposed side	No abnormality by naked eye
	Band-like or patchy subepithelial lymphocytic infiltrate	Massive cellular infiltrates of lymphocytes and neutrophils in the dermis	Diffuse inflammatory cell infiltrates consisted with CD5^+^, CD8^+^, and CD25^+^	Slight to intense lymphocytic subepithelial infiltrates	Patchy subepithelial infiltrates, including T cells
	Hydropic degeneration of basal cell with colloid bodies	Vacuolar degeneration of the basal layer with colloid bodies	No	No	Hydropic degeneration of basal/suprabasal cells with colloid bodies
	Intraepithelial lymphocytes	Lymphocytic invasion into the epidermis	Migration of activated helper T cells	No	Intraepithelial T cells, including CD4^+^ T
Construct Validity	Did the method used induce at least one face validity for OLP?	Yes	Yes	Yes	Yes
	Testing a specific hypothesis	A particular allo-MHC II-reactive CD4^+^ T cell clone induces LTR-like basal cell degeneration.	The F1 hybrid rat model of GvHD is useful to study oral mucosal lymphocyte-epithelial interaction but not OLP	Local toxicity of corrosion products rather than allergic mechanisms may contribute to amalgam-associated OLR.	*E. coli* infection can induce OLP-like histopathology.

## 4 OLL animal models in literature

Although no animal model for OLP currently exists, three OLL models are described in the literature.

Shiohara et al. presented a model where subcutaneous injection of an allo-Ia-reactive CD4^+^ T-cell clone into a mouse footpad induced hydropic degeneration of the basal cell layer and colloid body formation (11). Except the lack of oral lesions due to the injection site, this model meets three of four face validity criteria for an OLP model (Table 1). This study tested the hypothesis that helper T cells specific for class II MHC antigens are responsible for lichenoid tissue reaction (LTR). The authors could not answer why only one of three allo-Ia-reactive T cell clones induced LTR, despite all inducing massive dermal infiltrates of lymphoid cells and neutrophils. Reinterpretation of the data with current updated immunological knowledge suggests a role for the unique secretion of both IL-5 and IFNγ by the LTR-inducing clone, potentially inducing MMP-9 and MHC II expression in keratinocytes, respectively (15, 16). This implies that MMP-9-mediated basement membrane breakdown was necessary for T-cell invasion of the epidermis, and the degenerative interaction between T cells and keratinocytes depended on keratinocyte MHC II expression.

The second model involves GvHD induced by intravenous injection of spleen mononuclear cells from Lew (RT1^l^) rats into normal or irradiated Lew (RT1^l^)/Da (RTl^av1^) F1 rats (12). This study explored whether the rat F1 model of GvHD could induce an oral lichenoid reaction. While no overt oral mucosal abnormalities were observed, histology revealed diffuse subepithelial and intraepithelial infiltrates of CD5^+^, CD8^+^, and CD25^+^ cells in lingual oral mucosa, particularly in irradiated hosts, correlating with the GvHD index. However, hydropic degeneration was absent, despite the presence of activated (CD25^+^) cells in the epithelium and MHC II expression on keratinocytes. The authors concluded that the F1 hybrid rat model of GvHD is useful for studying oral mucosal lymphocyte-epithelial interaction but not for OLP.

The third model is an amalgam-associated OLCR in rats (13). Researchers tested whether contact allergic or local toxic effects contributeP to OLCR using mercury-sensitive (Brown Norway) and non-sensitive (Lewis) rats. Contact with amalgam or mercury-free amalgam alloy for 21 days caused development of blur- or plaque-like white lesions in the buccal mucosa of 97% of subjects. Histologically, these lesions correlated with slight to intense lymphocytic subepithelial infiltrates. The development of lesions was independent of the rat strain, alloy tested, mercury sensitization, or patch test results, while positive patch test reactions to amalgam or inorganic mercury were more frequent in the mercury-sensitized group (27% vs. 17%). Thus, authors concluded that the local toxicity of corrosion products, rather than allergic mechanisms, may contribute to amalgam-associated OLCR.

## 5 Recent advance presented at the 3rd international conference on oral mucosal immunity and microbiome

The hypothesis that bacteria infecting the basal cells of the oral epithelium and the lamina propria may induce OLP has previously been suggested (17, 18). Significant reductions in serum and/or saliva zinc levels have been observed in patients with OLP (19), and zinc supplementation has shown therapeutic benefits (20, 21). At the 3rd International Conference on Oral Mucosal Immunity and Microbiome, a new OLP mouse model developed by my research group was presented. This model tested the hypothesis that bacterial infection and zinc deficiency can induce OLP-like pathology (14). C57BL/6 mice, subjected to standard or zinc-deficient diets, received labial mucosal microdamage followed by oral administration of *Escehrichia coli* 7.2, a strain isolated from an OLP biopsy and detected in most OLP lesions (18). *E. coli* infection, in synergy with zinc deficiency or repeated epithelial microdamage, induced OLP-like histopathology in labial mucosa. The pathology was characterized by patchy subepithelial infiltrates, T cell recruitment into both the lamina propria and epithelium, hydropic degeneration of basal/parabasal cells with colloid bodies, increased saw-tooth-shaped rete pegs, and dissolution of the basement membrane. Therefore, this model satisfied three of four face validity for an OLP model. The synergy between *E. coli* infection and zinc-deficiency significantly decreased the Th1/Th17 ratio in the cervical lymph nodes and labial mucosa. Blocking Th1 cell differentiation during *E. coli* infection hindered bacterial clearance in the epithelium and exacerbated the histopathology, leading to subepithelial clefting. Collectively, this model demonstrated the contribution of infection with a specific microbe, such as *E. coli*, trauma, and trace element deficiency to the development of OLP-like histopathology.

## 6 Discussion

Here, various animal models to elucidate the pathogenesis of OLP and related disorders have been explored. While chronic GvHD and amalgam-associated OLCR have etiologies different from OLP, their clinical and histopathologic features overlap with those of OLP. Furthermore, the pathogenesis of chronic GvHD or amalgam-associated OLCR is not fully understood either. Therefore, the three OLL models described in the literature still hold significant value in clarifying the molecular mechanisms underlying the four face validities of OLP. For example, the allo-Ia-reactive CD4^+^ T-cell clone injection model could be instrumental in studying the role of donor CD4^+^ vs. host CD8^+^ T cells in the development of hydropic degeneration in chronic GvHD. It also can be used to identify crucial mediating cytokines or surface molecules using neutralizing antibodies or genetically modified mice. Insights from such studies could provide significant advances in understanding the mechanisms for hydropic degeneration observed in OLP.

Furthermore, our newly developed OLP mouse model offers a platform to study the roles of different T cell subsets, such as Th1, Th2, Th9, Th17, Treg, and CD8^+^ T cells, in the pathogenesis of OLP. This is especially important in the context of microbial infection and zinc deficiency, adding complexity to the disease pathogenesis. This model's ability to mimic key aspects of OLP pathology, combined with experimental manipulations, provides a unique opportunity to explore these complex immunological interactions.

It is important to recognize, however, that no single animal model can fully replicate the complexity of a human disease. The scarcity of OLP animal models is in stark contrast to conditions like psoriasis, for which 47 different animal models are available to study specific pathogenetic aspects (22). This highlights a gap in the research and underscores the need for continued development of diverse and sophisticated models to better understand and treat complex diseases like OLP.

In conclusion, while these animal models provide valuable insights into OLP and related disorders, they also highlight the need for ongoing research. The development of more comprehensive models that can accurately reflect human conditions is crucial. Such advancements will not only enhance our understanding of the pathogenesis of OLP but also pave the way for more effective and targeted treatments, ultimately benefiting those who suffer from this uncurable disease.
